# Crisis and Intergovernmental Retrenchment in the European Union? Framing the EU’s Answer to the COVID-19 Pandemic

**DOI:** 10.1007/s41111-020-00171-0

**Published:** 2020-12-28

**Authors:** Eugenio Salvati

**Affiliations:** Department of Economics and Management, University of Pavia, Via San Felice, 5, 27100 Pavia, PV Italy

**Keywords:** European union, European integration, COVID-19, Disintegration, Intergovernmentalism

## Abstract

The outbreak of the COVID-19 pandemic has placed severe pressure on the EU’s capacity to provide a timely and coordinated response capable of curbing the pandemic’s disastrous economic and social effects on EU member states. In this situation, the supranational institutions and their models of action are evidently under pressure, seeming incapable of leading the EU out of the stormy waters of the present crisis. The article frames the first months of management of the COVID-19 crisis at EU level as characterised by the limited increase in the level of steering capacity by supranational institutions, due to the reaffirmed centrality of the intergovernmental option. To explain this situation, the article considers the absence of the institutional capacity/legitimacy to extract resources from society(ies), and the subsequent impossibility of guaranteeing an effective and autonomous process of political (re)distribution, the key factors accounting for the weakness of vertical political integration in the response to the COVID-19 challenge. This explains why during the COVID-19 crisis as well, the pattern followed by the EU is rather similar to past patterns, thus confirming that this has fed retrenchment aimed at the enforcement of the intergovernmental model and the defence of the most sensitive core state powers against inference from supranational EU institutions.

## Introduction

In the last 10 years, the European Union (EU) has been through several crises which have led to the questioning of the trajectory of the EU’s integration process. These crises have raised the question of how a complex institutional structure devoted to pursuing strict regional integration—that is, an increasingly closer Union—based on a routinized, institutionalised model of strict cooperation, could possibly fail to deploy the invaluable institutional instruments enabling it to navigate these choppy waters, without (or severely minimising) the risk of stalemate, or indeed of the disintegration of the Union. The outbreak of the Covid-19 pandemic is putting under stress world’s political, economic and social systems implying an unavoidable reconfiguration of the post-pandemic international order (Huang 2020).

This raises certain preliminary questions regarding how to account for the way in which the EU is coping with the COVID-19 crisis, and its impact on Europe’s economies and societies. How are we to frame and explain the decisions taken by the EU? Will the response to the pandemic favour a move towards a more supranational EU, or will it help enforce the intergovernmental dynamic?

Despite the fact that we are in the amidst of the pandemic, and are reflecting on a dynamic and processual dimension instead of a well-defined political outcome, the article will analyse the first decisions taken by the EU and try to frame the type of response deployed and its potential consequences for the EU. The article argues that despite the Commission’s considerable activism, the response coming from the EU can be seen as confirmation of the difficulty experienced in further raising the level of integration (vertical political integration) by enforcing the role of supranational institutions during a crisis. This outcome can be accounted for by the EU’s inability to forge its own autonomous capacity to extract resources from European society(ies), and thus to enforce a coordinated, supranational response. This limit unavoidably produces a process of crisis management, which comes close to vertical political disintegration as a result of the enforcement of the intergovernmental decision-making model.

## Defining the Integration Trajectory

The COVID-19 pandemic has hit in the aftermath of several other crises such as the Eurozone, migration/Schengen and Brexit crises, which have seriously threatened the very essence of the EU by triggering the risk of the effective disintegration of the European Union (Webber 2019). Despite these consecutive structural threats, the EU has shown that it represents a very resilient project for regional integration and has managed to curb the risk of disintegration by pursuing a new equilibrium and relying more heavily on intergovernmental powers, resources and decisional models (Fabbrini 2019; Hodson and Puetter 2019).

Unlike the other crises experienced over the last decade, the Coronavirus pandemic cannot be claimed to be “structural” since it does not directly involve the scope of integration, the functioning of European institutions or put into question the membership of some member states. Furthermore, it impinges on a sector, that of healthcare, that is a policy field over which the EU has very limited powers. Nevertheless, the pandemic crisis is triggering severe tensions which are putting under stress the relationships among states as witnessed, for example, by the escalated conflict between US and China over the origin and the management of the virus (Jaworsky and Qiaoan 2020).

For this reason, a reflection regarding the impact of COVID-19 on EU politics is extremely important, since the pandemic outbreak is posing a series of threats that are in any case severely challenging the EU’s crisis management capacity and readiness, since this specific crisis indirectly involves key aspects of the integration process. I refer in particular to the risk of supranational institutions becoming highly marginal and/or weakened vis-à-vis intergovernmental institutions to attenuate the impact of the pandemic on the European economy and the macroeconomic shocks, and to the consequent difficulty of triggering political solidarity and effective burden-sharing among member states. These difficulties can hamper the level of political integration and so lead towards a stalemate and/or vertical political disintegration. Finally, another peculiar aspect involved is the complicated endeavour to define permanent institutional instruments capable of enabling the EU to react directly and in a timely fashion to different crises, while minimising the bargaining and opting-out risks. All of these elements, regardless of whether the crisis has not been provoked by, or is traceable to, EU-level institutional and policy mechanisms are straining the EU’s ability to provide a timely, coordinated, “European” answer to the severe socio-economic consequences of the pandemic.

Up until the outbreak of the Eurozone and Greek crises, EU integration was conceived as a successful and (probably) almost completed process (Moravcsik 2005) and the risk of stalemate and/or disintegration was not considered credible. Within the context of EU integration, the broadening and nature of institutional construction, together with its stability and effectiveness, pointed to the unlikeliness of such worrying outcomes.

The main shortcoming of this vision was probably the binary conception of integration/disintegration that fails to take into consideration the eventuality that this mechanism may operates within a political system. In a political system, the processes of disintegration in certain sectors may occur concurrently with moves towards integration in other areas; the reason for this is that disintegration is not simply the reverse of integration (Vollaard 2014).

If we go back to the roots of integration, Haas conceived it as a process “whereby political actors in several, distinct national settings are persuaded to shift their loyalties, expectations and political activities toward a new centre, whose institutions possess or demand jurisdiction over pre-existing national states” (Haas 1958, 16).

Following Haas’s idea of the expansion—or reduction—of issues managed at European level and the role of the EU vis-à-vis the member states, Webber (2019, pp. 13–14) frames the different shapes that integration/disintegration could take. He portrays horizontal (dis)integration as the increase or reduction in the number of member states that are part of the Union. Sectoral (dis)integration, on the other hand, concerns the expansion or reduction in the scope of EU policy action and the number of policy issues on which the EU has competences. Finally, he sees vertical political (dis)integration as the expansion/reduction of the EU’s official competences and political authority wielded by supranational institutions and the increasing power of the intergovernmental method and organs. This dimension is the one that concerns this study, since it identifies the so-called level of EU integration (Börzel 2005). This level is particularly low when the EU acts through less intrusive modes of policy formulation preserving core state powers (Héritier 2012). This condition avoids the risk that the EU may develop a centralised exercise of power contrary to that of its member states and implies as a connected outcome the enforcement of the intergovernmental arena. The limited nature of integration, the possible occurrence of vertical political disintegration and the centrality of the intergovernmental model are precisely what seem to characterise the pandemic crisis.

In this shape, the enforcement of vertical political integration at EU level can be operationalized also as the definition of new (formal and informal) rules which modify the status quo and strengthen the role of supranational institutions at the expense of the Council and of EU member states, giving more power to weaker institutions and so raising the level (and scope) of integration (Stacey 2010).

Given such a complex framework, the evolution of EU integration can take different paths under the label of ‘differentiated integration’ (Schimmelfennig et al. 2015), which would explain the institutionalisation of models of graded, unequal and differentiated membership. According to Schimmelfennig et al. (2015), differentiation is a permanent structural feature of the EU, and is the result of the interaction of interdependence with politicisation. Said authors distinguish two types of differentiation (2015, p. 765): vertical differentiation, which refers to “policy areas that have been integrated at different speeds and reached different levels of centralization over time”; and horizontal differentiation, which “relates to the territorial dimension” of integration. This model of integration is seen as a shield against the exercise of the EU’s power and authority to the detriment of member states’ sovereignty, thus institutionalising a core-periphery divide (Schimmelfennig and Winzen 2014) useful to the defence of national interests and identities, also in response to the pressure of public opinion and of nationalistic, Eurosceptic parties (Schimmelfennig 2018; Fabbrini 2019; Hooghe and Marks 2019).

If differentiated integration is a useful option preventing the defection or obstructionism of countries that are unwilling to cooperate, on the other hand it may weaken compliance with the provisions of EU law by undermining the norms of legal unity (Schimmelfennig and Winzen 2014), and by creating a fragmented institutional system based on a complex model of multiple arenas for cooperation that prevents the achievement of a more compact, efficient form of integration. Nevertheless, as Schimmelfennig (2018) explains, even the existence of a model of differentiated integration does not prevent the explosion of differentiated disintegration, as has been seen in the case of Brexit.

The interaction of models of (dis)integration and differentiated integration, offers a complex, multifaceted picture, where different features of the EU political system’s architecture contribute towards curbing the likelihood of a more centralised authority at supranational level.

If we take into consideration certain parameters of a political system’s integration, it is easy to see how a model of differentiation can weaken integration and feed various different dynamics of disintegration. The integration of a political system can be measured by the degree to which:

- It can guarantee compliance with the norms and rules it establishes;
- It can extract resources from society and control the allocation, distribution and redistribution of these resources;
- It is a benchmark for the identification of a political community.

Likewise, the risk of stalemate or disintegration can be considered as existing when the degree of control over the aforesaid three aspects is particularly weak, is questioned, is the subject of bargaining, or is ultimately reduced. A system which relies heavily on multiple opportunities for differentiated integration is more likely to weaken these three elements, thus preventing effective integration and facilitating horizontal and/or vertical disintegration.

The possible outcomes could consist of: apathy in the integration process, which would point towards a stalemate or decision-making models far removed from an integrationist logic (Kelemen 2007); the definition of suboptimal integration options (Jones et al. 2016); differentiated disintegration (Schimmelfennig 2018); or even pure disintegration (Schmitter and Lefkofridi 2016; Webber 2019).

It is worth pointing out that stalemate and/or disintegration occurs when there is a substantial shift of power from less powerful to more powerful institutions (Stacey 2010). For instance, in the case of the prevalence of the European Council and the Council of the European Union over both the Commission and the European Parliament (Stacey 2010; Fabbrini and Puetter 2016).

From this point of view, it is difficult not to perceive the course of the various EU crises as constituting a pattern of vertical disintegration—and a step backwards from the community method—due to the marginalisation of the Commission and the EP in favour of the resurgence of member states and the intergovernmental method (Börzel 2005; Fabbrini 2013, 2019; Webber 2019). Despite the inherent differences between the COVID-19 crisis and the main crises which the EU has gone through in recent years, can the initial response to the pandemic be seen as representing a degree of continuity with the dynamics of vertical disintegration? How can we explain the risk of vertical disintegration and the dominance of the intergovernmental option also in relation to the COVID-19 crisis?

Despite the Commission’s attempt to lead the response to the pandemic, the intergovernmental option has proven the main steering force, and as we will see in the empirical section of the article, member states appear ready to cooperate and reduce the risk of a crisis of the EU polity, but are also keen on avoiding a response that is excessively centralised in supranational hands. The article’s theoretical argument is that the answer lies in the inability of supranational institutions to independently extract resources from European society(ies), and in the corresponding difficulty experienced by supranational institutions in infringing intergovernmental defence of certain specific core state powers. This is the missing piece of the puzzle concerning the level of EU integration and the risk of stalemate/disintegration, which needs to be included to better understand the EU’s response to the COVID-19 challenge.

## The Missing Piece

The aforementioned elements provide a framework with which to explain the EU’s complex reactions to different crises, and the fluctuation between supranationalism on the one hand, and intergovernmentalism on the other, and between different levels of integration and the risks of vertical disintegration. In this respect, there is a missing piece that can complete the picture and give us a more comprehensive and fully-fledged theoretical framework.

This “missing piece” can be found in that part of the literature on the EU that focuses on the formation of this new political centre, through the EU’s system building and political structuring capacities (Bartolini 2005) and the related dynamics of potential integration/disintegration (Vollaard 2014). These political and community building dynamics are of the utmost importance, since they impinge on how the coordination/competition between subunits and the political centre unfolds within a context of multilevel governance (Jachtenfuchs and Kasack 2017), and how this new supranational political entity tries to influence, and encroach on, traditional core state powers (Genschel and Jachtenfuchs 2016), triggering member states to take a defensive stance which retrenches on intergovernmental models of decision making.

The starting point for the said analysis is Bartolini’s work (2005), which identifies European integration as the last stage in the lengthy development of the European states’ system. According to Bartolini, EU integration is based on three distinct, yet intertwined, processes:

1. The formation of a would-be political centre in Brussels possessing marginal political authority;
2. A process of supranational system building which is producing weak institutional mechanisms capable of uniting the different actors in this new system;
3. A rather incomplete political structure for the channelling of conflict dynamics.

One of the main problems with this impaired process is that the EU possesses virtually no basic capabilities when it comes to political structuring and system building. This deficiency has created a spiral of de-structuring of member states’ boundaries through the process of centralization, at EU level, of a substantial series of powers and functions, without the corresponding structuring of new legitimation and representation mechanisms at the supranational level (boundary building). This condition specifies a rather weak lock in power at the EU level that translates dissatisfaction with the EU into partial exits which cannot be converted into voice options or full exit choices (Vollaard 2014). Such a complex conjunction of structural imperfections and drawbacks may account for the process(es) of EU stalemate and disintegration.

The condition drawn by Vollaard, in which resources and actors cannot be fully locked in within an incomplete political system, may be accounted for by a structural lack of power and authority that affects the EU. The EU’s main problem is not simply the incapability to lock in resources but rather its inability to autonomously and directly increase and mobilise its own resources from society(ies).

The occurrence of a crisis like the COVID-19 pandemic requires the centre of political power to possess the organisational and political tools permitting it to autonomously obtain the resources it needs to face such a challenge, and thus implement policies that are of value to all parts of the political system. The opportunity to extract different types of resources from society(ies) (economic, symbolic, and legitimacy) is essential to effectively exercise executive power and authority over a given territory.

On the one hand, if we consider the context of the EU and its specific features, it would be naïve to think of an extractive model fully comparable to the nation state that embraces properties like the preservation of internal and external security, exclusive taxation power, complete fiscal harmonisation, and so on. On the other hand, the degree of interdependence among member states, the number of competencies devolved to the EU and the common perspective defined by a severe crisis, would together require an independent, centralised, supranational capacity to act to preserve the good functioning of the political centre and its legitimacy. This is an essential condition if fast and effective answers are to be provided to new needs and risks which, if managed in diverse ways could produce ineffective results, feed disparities among partners, and potentially trigger the dynamics of disintegration.

The EU does not possess such powers, faculties and consequent institutional instruments. Instead, what characterises this “would-be political centre” is its extreme internal fragmentation: the more the parts constituting the Union are substantially autonomous in managing a crisis, the more complicated, if not impossible, will be for the central/supranational authority the opportunity to create (or implement) an independent extractive power. This will be the case until the resources available at EU level, and the use of such resources, are the result of a process of mediation by the multiplicity of actors involved. The EU does not exercise independent extractive powers, but merely provides an institutionalised bargaining arena in which different states coordinate their resources’ mobilisation capacity within a common framework (Genschel and Jachtenfuchs 2016). The EU has undeniably taken over certain core state powers in recent years (particularly following the emergence of severe crises). However, this dynamic has been possible due to the intergovernmental choices made by EU politics, and, moreover, has contributed to the increase in political tension and to policy fragmentation by fuelling the conflict between Eurosceptic and Europhile positions, and by segmenting EU policies as a result of conflict between national publics (Genschel and Jachtenfuchs 2016; Jachtenfuchs and Kasack 2017). Furthermore, the EU still lacks a form of political legitimacy for its claim to be equipped with such powers, resources and functions, in a contest defined by an inherent tension between national and supranational configurations (Bolleyer and Reh 2012).

This state of affairs is further complicated by the fact that during every crisis, the EU establishes different tools and institutional instruments to deal with that specific crisis, thus resulting in an astonishing degree of institutional proliferation (Genschel and Jachtenfuchs 2016; Schmitter and Lefkofridi 2016) which while providing a short-term problem-solving capacity on the one hand, on the other weakens the EU’s ability to create stable instruments with which to quickly and effectively cope with different threats.

Consequently, the more fragmented the institutional and decision-making landscape, the more the actors’ coordination becomes difficult, resulting in the risk of partial (differentiated integration) or total (horizontal political disintegration) exits, in a reduction in the level of integration (vertical political disintegration), and in the settlement of suboptimal agreements which lead towards less effective coordination (Jones et al. 2016).

Paramount to this process is the fact that the attempt to mitigate the effect of fragmentation can only be pursued by means of the intergovernmental option. A key aspect of this process of (formal and informal) institutional design is that this dynamic unavoidably implies a redistribution of power among EU actors. Even if these agreements may in the short run result in interdependence, this does not mean that they favour more supranational integration or increase the level of integration, while vice-versa, are instrumental to the strengthening of the member states’ role and the intergovernmental approach (Stacey 2010). This situation results in a course of action whereby supranational institutions (in particular the European Commission) are not merely deprived of any decisional role, but are indeed relegated to the role of executors and overseers of intergovernmental agreements (Bressanelli and Chelotti 2016; Fabbrini and Puetter 2016; Webber 2019).

Within such a framework, where the “new political centre” has no effective power or authority to take key decisions binding on all partners, a hegemonic leadership may emerge as an important solution capable of avoiding the risk of disintegration (Webber 2019). Different, variable alliances among actors may coalesce around this leadership, capable of achieving a political agreement. As a rule, in a certain way such agreements foster a higher degree of interdependence among actors, thus resulting in an outcome that is suboptimal compared to effective needs, and which, more importantly, does not provide any institutionalised instrument capable of resolving any further crises.

All these elements determine partial or incomplete progress; however, this is enough to lay the foundations for more comprehensive solutions in the future, following the dynamic that Jones et al. (2016) have referred to as ‘failing forward’. Using this concept, Jones et al. contend that European integration can proceed through cycles of integration based on the lowest common denominator, that is, agreements which result in incomplete institutions, which in turn generate crises that imply a shift towards a deeper form of integration through incomplete institutions. The incompleteness of these institutions is so significant that this can feed other political crises, which in turn threaten to destroy the integration process. To avoid this collapse, member states governments, and not EU supranational institutions, agree to establish the further minimal reforms required to save their gains in the short term, but this still leaves institutions incomplete in ways that will subsequently engender another series of crises and reforms. This dynamic allows the process to repeat itself.

Such an approach in critical areas can provide short-term or medium-term solutions, but is incapable of dealing with such critical aspects as the lack of solidarity and the absence of powerful centralised institutions.

In a nutshell, the answer cannot be purely (or mainly) of a supranational nature, due to two interconnected features: the absence of the institutional tools and political legitimacy permitting the independent extraction of resources by EU institutions; and the fact that in the absence of such centralised arrangements, the use of resources is decided exclusively through intergovernmental bargaining (which can produce suboptimal agreements and incomplete institutions).

The decision to keep these areas and integration paths eminently intergovernmental is related to the question of “who pays” and “who benefits”, with member states needing to preserve control over resource allocation and redistribution, means that this process is perceived more in national than in European terms (Genschel and Jachtenfuchs 2016). As the Eurozone crisis has shown, and as partially reiterated in the early stages of the COVID-19 crisis, countries are not necessarily willing to move towards deeper integration since they refuse to accept cross-national resource transfer and redistribution policies (Beramendi 2012), or they try to avoid the connected moral hazard risk.

The push towards intergovernmental answers guaranteeing the limited role and pervasiveness of supranational arrangements has been influenced also by pressure from national political systems. Eurosceptic parties and those sections of the public critical of the EU are able to create an internal constraint on mainstream parties and national governments, designed to curb their willingness to increase the level of integration. This state of affairs is accounted for by post-functionalist theorists who focus on the role of political conflict and identity politics within national political systems, and their impact on the integration process (Hooghe and Marks 2019). The shift of a degree of political decision-making authority away from nation states is identified as the paramount obstacle to the preservation of national identity, the defence of national governments’ capacity to intervene in the economy, and the related capability to safeguard national authority. This hostility is exacerbated by the de-alignment between administrative and functional borders that is produced by EU integration (Bartolini 2005) and by the fact that political competition and mass politics (with their cycle of responsiveness-representation-accountability) are encompassed within national borders, while a large part of political production that is centred in Brussels, thus undermining the connection between political trust and legitimacy and problem-solving capacity (Salvati and Vercesi 2019).

All these factors contribute towards reshaping national party systems, stimulating the rise of populist and Eurosceptic parties that politicise the pro/anti-EU cleavages, and polarising political proposals in terms of more radical stances hostile to greater supranational EU integration (Grande and Kriesi 2016; Salvati and Vercesi 2019), on the basis of forceful demands for the defence of national sovereignty in core sectors of state power (Schimmelfennig 2018).

In a situation where the political centre’s lack of resources, instruments and legitimacy with which to provide prompt solutions, and where national governments are under pressure from public opinion to reduce or limit the level of integration, problem solving can only be addressed by the constitutive actors of this supranational polity, that is, by the member states themselves.

The EU context, particularly following the Eurozone crisis, is characterised by a low level of mutual trust and solidarity (Jones 2012; Ferrera 2017) typical of a rescaling process (Keating 2013), with multiple cleavages that nourish different—(re)distributive and on rule of law—(North vs. South, East vs. West), fed by national public opinion which seems keener to reject greater integration and inter-member state solidarity, in favour of more nationalistic (Fabbrini 2019) or even regional (Keating 2013) options. Given this situation, it is not surprising that support for mechanisms of interstate distribution and redistribution of resources is rather weak and is mainly subject to conditionality mechanisms of some kind. As shown by the evolution of institutional performance within the EU over the last decade, the real issue is not the presence or absence of conditionality in the definition of new institutions/policy instruments, but rather the degree of such conditionality.

## The COVID-19 Crisis and the EU’s Response. A Turning Point or a Risk of Stalemate/Disintegration?

Having defined the framework, it is now possible to analyse how the EU managed the COVID-19 crisis during the early stage of the pandemic. Are there any empirical signs pointing to limits in the level of integration in the response offered to the crisis, and to the prevalence of an intergovernmental approach instead?

It is important to apply certain caveats to this analysis, since the pandemic is still on going, and we only have partial data/information, so we cannot determine what final path the EU and member states will take and what the final political outcomes will be. Nevertheless, certain preliminary insights can be offered into the initial responses provided, together with a preliminary reflection regarding their implications.

The EU’s reaction to the pandemic can be defined as significantly differentiated over the course of the first 4 months, consisting of a fragmented response at multiple levels and among diverse actors. Indeed, member states have refused to recognise the Commission’s role as the coordinator of individual national strategies (as seen in the failure of the Joint Roadmap for lifting COVID-19 containment measures). The timeliest substantial action has been that taken by the European Central Bank (ECB), which is the only supranational institution that can offer a rapid response to the crisis and effectively mutualize the risks involved. The ECB deployed its Pandemic Emergency Purchase Programme (PEPP), a temporary measure designed to ensure supportive financing for all sectors of the economy (Lagarde 2020). The PEPP consisted of an initial amount of €750 billion, which was later expanded to €1,350 billion and extended until the end of June 2021, or until the Governing Council estimates that the coronavirus crisis is over (ECB 2020).

This decisive action has been accompanied by the Commission’s decision to permit the flexibility of state aid rules that restrict individual government’s ability to subsidise companies, and to temporarily interrupt the stability and growth pact (SGP). While this decision enables national governments to independently organise their initial responses to the crisis, in the medium-long term this decision, together with a limited degree of coordination, could increase asymmetries among member states due to their different fiscal space (Fleming and Espinoza 2020). Member states with greater debt-bearing capacities and more resources to spend (i.e. Germany, Austria, and the Netherlands) will gain an unfair advantage over those member states with more limited resources (i.e. Spain, Portugal, and Italy), thus skewing the functioning of the single market and the level of integration.

From a certain point of view, the Commission’s decision is of historical importance since it represents a departure from a principle of strict fiscal discipline in the EU; on the other hand, however, it confirms the structural deficit we were discussing, that is, the impossibility for EU supranational institutions to autonomously extract resources. This lack of power and authority translates into the inability to directly use its own resources to respond to the pandemic crisis in a timely and effective manner. The Commission’s only opportunity to offer a response and not leave the entire burden of action on the European Central Bank’s shoulders, has been to unleash the member states’ capacity to spend; however, this comes at the risk of endangering the very meaning and functioning of the common market (Fleming and Espinoza 2020) and of potentially triggering sectoral disintegration.

Likewise, the other main supranational institution—the European Parliament—has been even more marginalised during this phase, thus confirming its peripheral role during major crises (as was the case in the Eurozone crisis). Its only action has consisted in approving a non-binding resolution in favour of a recovery plan included within the Multiannual Financial Framework (MFF). The connection with the MFF is the only way for the EP to have a say in the economic management of the crisis, and to underline the belief that the distribution, allocation and screening of resources should be a political matter rather than a technical one.

Amid this change, the member states’ governments have thrown themselves into the battle concerning the sharing of sovereign debts by means of the so-called coronabonds. The idea of mutualising debts and dealing with the costs of the pandemic in a supranational way has been put forward by France, Italy and Spain, who in a letter signed by a further six states, openly called for this solution to be adopted to overcome the difficulty experienced by some states in getting access to credit markets and borrowing money at sustainable costs.

This proposal, which opened the way for a lengthy series of intergovernmental negotiations, has been rejected—in a first step—by the Northern European states, led by Germany and the Netherlands, who cannot accept the risk of encouraging moral hazard or fiscal irresponsibility, due to a strong, deep-rooted hostility within their political systems towards any kind of resource redistribution and cross-national transfers from North to South (Statham and Trenz 2015; Hobolt and De Vries 2016; Ferrera 2020).

Following the same path established during the Euro crisis, in the first weeks after the end of the crisis a number of Northern European countries pushed for the use of the established European Stability Mechanism (ESM) as the best (and only) instrument for indebted Southern European countries to borrow money and use it for the purposes of crisis management. This would lead to the application of strict conditionality measures—reforms and macroeconomic adjustments—which would be unacceptable to Spain and Italy (Valero 2020).

To mediate between these two distant positions, the Eurogroup decided to approve the first package of three measures designed to constitute the first European response to COVID-19 (Eurogroup 2020). The first decision was to back the Commission loan-based programme for sustaining national insurance systems, the SURE action (Support to mitigate unemployment risks in an emergency).

The Eurogroup also approved two intergovernmental measures: a European Investment Bank programme supporting lending to small and medium-sized enterprise, and another measure by the European Stability Mechanism—the Pandemic Crisis Support (PCS)—designed to make loans available to national governments to pay exclusively for health care expenses related to the pandemic (determining, with this bond, a sort of light conditionality) under the supervision of the Commission, for a period strictly related to the duration of the COVID-19 crisis.

The SURE fund is of particular importance due to its capacity to issue loans of up to €100 billion aimed at financing short-term employment schemes. To do so, Member States will have to provide national guarantees of up to €25 billion, which will be used by the Commission to issue triple-A bonds which are then turned over to the Member States through long-term loans. In a nutshell, this can be seen as the first sort of “Eurobond” with a form of joint debt (member states jointly issue the debts through the Commission and pay them back jointly). This aspect should not be underestimated since it constitutes a significant precedent, and is especially meaningful for those countries that are against any form of debts mutualisation. What is interesting about the SURE mechanism is that it raises the level of interdependence and integration among partners. The limitation of this scheme is that being subjected to pressure from those countries most hostile to any form of debt mutualisation (the so-called ‘frugal states’), it can only be of a temporary nature and does not represent the core of a European unemployment insurance scheme, thus substantially hampering its supranational impact. Furthermore, despite the Commission’s ambitions, the amount available is a lot less than the member states need.

The agreement on the SURE fund, together with the suspension of the SGP mechanism, can be considered to be the main tangible results of the Commission’s efforts to directly manage the impact of the crisis on member states and the credibility of EU institutions. These efforts represent the Commission’s attempt to foster a European response to the crisis by establishing a supranational set of measures with which to curb the pandemic’s macroeconomic effects. They are designed to reduce the risk of stalemate and vertical disintegration. Unfortunately, the strength of the SURE response has proven extremely limited, and the suspension of the SGP is more a way of “giving back power” to national governments than of using direct supranational authority.

Despite these efforts, the main pillar of the EU’s response to the COVID crisis is represented by the NextGenerationEU plan (Herszenhorn et al. 2020). This is a proposal for a new recovery fund of €750 billion linked to the new MFF, based on a mix of grants and loans which will boost the EU budget with an injection of new resources raised on the financial markets over the 2021–2024 period, with bonds issued at maturities extending to 2058 (European Commission 2020). Despite its reduction from the amount established in the Commission’s initial draft plan, €390 billion of the €750bn will be distributed as grants, and therefore, will not add to governments’ debt burden: this represents a real break from the open hostility to substantial intra-EU fiscal transfers (European Council 2020). The resources will be allocated to member states in proportion to the impact that the crisis is having on each of them.

Such a plan, although presented by the commission, can hardly be considered the result of a pure supranational effort, for at least two reasons. On the one hand, its scope and operation have been heavily influenced by the Franco-German recovery plan proposal and by the new orientation of the Merkel’s government; on the other hand, it has been, and will be, subject to strict negotiation among EU heads of state and governments, and requires unanimity at Council level.

In this regard, the action of the so-called “frugal states”, that is, Austria, Sweden, the Netherlands, Finland and Denmark, has led to the introduction of substantial conditionality to the plan. Member states wishing to access funds will have to present investment plans, and these plans will be evaluated in accordance with the country recommendation annually delivered within the framework of the European semester. More important is the fact that due to pressure from the Dutch Prime Minister Mark Rutte, an “emergency brake” has been included, meaning that any government can object to another’s spending plans, thus delaying and complicating disbursements. This option offers some important insights into the path taken by the crisis management process. First of all, some governments do not completely trust the Commission’s technocrats and their ability to effectively scrutinise and supervise the use that other governments may make of the available resources. This implies that the steering of the response is not in supranational hands. Secondly, this governance model implies that in the management of the response to the shock produced by the pandemic, there is a persistent (at least partial) lack of real mutual trust among member states. Finally, and probably most importantly of all, this arrangement permits the most critical national governments to publicly claim that they still have control over funds and pooled resources.

Furthermore, questions remain about whether this course of action will see supranational dynamics prevail within the EU, due to the fact that the plan is of a temporary nature and does not necessarily set a precedent for future crises. Moreover, and most important according to our framework, member states continue to be strongly reticent to the idea that the Commission extensively finances this disbursement of funds by deciding to levy EU-level taxes. A decision that should be ratified by all EU member states in line with their constitutional requirements, so giving space to other possible political oppositions.

Finally, agreement on the plan has been reached subject to other important concessions being made to the “frugal states”. For example, they have obtained a considerable reduction in the budget allocation for the so-called “EU future-oriented” programmes concerning research, health care and climate adjustment. These are all areas in which the supranational orientation of the related policies is particularly evident; this gain underlines the goal to indirectly reduce the EU capacity to steers member states decisions in such relevant sectors. Furthermore, the frugal have been granted considerable increases in the rebates they get on their EU budget payments, thus further complicating the difficult negotiations awaiting the next MFF. From this point of view, the need for a strong agreement among member states (and the subsequent need for approval from each and every member state’s parliament), shows that the most important part of the political response to the crisis will be forged once again by the intergovernmental model, thus avoiding the risk of enforcement by, and the empowerment of, the political centre in Brussels. This is very clear if we consider that at the present moment in time, the only financial instrument available to national governments is the PCS, which is a product of intergovernmental negotiation within the Eurogroup.

One of the most significant aspects of the genesis of the plan, regardless of its content, is the fact that it is both directly and indirectly the product of the German government’s rethinking of its position vis-à-vis the instruments to be deployed to manage the crisis. After an initial period of resistance to the adoption of instruments similar to Euro bonds, Angela Merkel reconsidered her government’s position by assuming a more conciliatory stance regarding the requests made by the countries severely hit the crisis.

This rethinking led the German government to change its approach towards the economically weaker countries from that adopted during the Eurozone crisis. This change can be seen in intergovernmental terms, as a new way of protecting German interests in Europe, since the considerable degree of issue interdependence has boosted the significant political reorientation of the German government. For example, the need to sustain a large economy such as that of Italy, which is highly integrated with the German economy (Mallet et al. 2020), means safeguarding the German productive chain and the integrity of the single market. Furthermore, this reckoning is also the German government’s response to the highly-debated ruling by the German Constitutional Court, which established that the ECB’s public sector bond purchases may be ultra vires, or unconstitutional. The Court ordered that the government and parliament in Berlin ensure that the ECB carried out a “proportionality assessment” of the bond-buying process to ensure that its “economic and fiscal policy effects” did not outweigh other policy objectives (Arnold 2020). Part of the German public, together with diverse players within the German party system—like the Euro critical wings of the Christlich Demokratische Union Deutschlands (CDU), Christlich-Soziale Union (CSU) and the right-wing populist party Alternative für Deutschland*—*see this ruling as an attempt to prevent Germany’s political power being eroded by Brussels, and stop its wealth being used to bail out Southern European countries.

The centrality of the political positions adopted by the German government, in agreement with that of France, confirms the possible return of a structural Franco-German coalition that could in turn imply a form of hegemonic stability designed to reduce the risks of stalemate and/or disintegration (Webber 2019). The withdrawal of the UK from the EU following the Brexit referendum and the absence of a stable, permanent coalition among Southern countries,^1^ reveals a situation in which the gap in strategic powers will be greater than in the past, thus entail the likelihood that this outcome will lead towards the dominance of the two most powerful actors, rather than mere hegemonic stability, which in turn raises doubts as to whether the final result is going to be more effective integration under the label of stability, or whether this dynamic will produce a stalemate that could feed apathy and disintegration under an illusory form of political stability.

In any case, the underlying concept of a NextGenerationEU does not imply a complete refusal of the principles of control and rigour of public budgets, since the plan in question involves the disbursement of resources being linked to a mechanism of constant evaluation and monitoring of the member states’ projects submitted to the Commission and the Council, within a rigorous framework of rules defined by the European Semester.

This is particularly important given that the initial period of this crisis has confirmed the presence of considerable hostility towards any form of transnational resources redistribution and “would-be” debts mutualisation. I refer to the axis of the so-called “frugal states” at the forefront of this battle: this group of countries demand that strict conditions be applied to any fund disbursement among member states, together with a reduction in the total funds to be made available as grants. In this case, the governments in question are having to deal with pressure from Eurosceptic and nationalist parties, which are against any form of mutualisation or strong inter-EU solidarity. This is why these countries demand a drastic reduction in the share of the fund to be assigned through grants, and that these instruments—like the SURE or NextGenerationEU—will be of a temporary nature only. Furthermore, these countries oppose a further two key questions: an increase in the EU budget, and providing the EU with its own taxation capacity (i.e. digital tax and taxation on emissions). These disputed questions reveal the resistance to giving the EU room to autonomously extract resources, and thus set a precedent that could result in the EU having autonomous “tax and spend” powers.

Furthermore, there is another line of conflict: the threat of a veto being exercised on European Council decisions by the Hungarian and Polish governments, both of which refuse any kind of connection between the MFF and the disbursement of recovery funds on the one hand, and the respect for civil and democratic rights in their own countries on the other.

## Discussion and Conclusion

In this article, I have tried to illustrate the early steps taken by the EU in response to the pandemic crisis and its consequences. This response has been mixed (with both supranational and intergovernmental actions), innovative (for certain aspects), complex and fragmented. The incapacity of EU supranational institutions to exercise the function of resource extraction is key to accounting for the impossibility of any autonomous, genuinely supranational response also in the case of the COVID crisis. This lack of power and authority deprives the EU of the opportunity to have a political production able to deploy quick and effective (re)distributive instruments designed to absorb internal and external shocks.

Such an arrangement is confirmed by the limitedness of the anti-crisis provisions, which can be directly ascribed to supranational institutions like the Commission and the EP: we have a limited amount of resources in macroeconomic terms, that are strictly constrained in scope, not readily available to governments and limited in their duration. Moreover, the bitter conflict among the Commission and the governments about which instruments to use to manage the COVID-19 crisis, have emphasised and confirmed the “unrealistic character of the state like approach pursued by supranational actors, but also the disintegration’s implications of the multiple “statenesses” approach pursued by national leaders” (Fabbrini 2019, p. 489). This dynamic shows that when the EU needs to focus more on core state powers to resolve significant crises, member states are keener to develop (new) intergovernmental structures and tools than to accept the greater influence and authority of supranational actors (Genschel and Jachtenfuchs 2014; Jachtenfuchs and Kasack 2017), thus triggering a process of vertical political disintegration as a result of constraints hampering the level of integration. Contextually, the intergovernmental option allows to limit the risks of sectoral (and even horizontal) disintegration.

The only exception to this marginalisation of supranational institutions is represented by the ECB, which is a technocratic, non-majoritarian institution. Its indispensable activism, which is timely and of vital importance in providing a strong economic response to the crisis, underlines the weakness of EU politics and the existence of the permanent difficulty in coordinating (also at intergovernmental level) quick and effective responses. The risks of stalemate and disintegration are essentially being contained by the ECB’s direct, prompt action which provides a monetary umbrella to member states. However, this again is a political response that has been delegated to technocratic authority, which whose actions are not subject to democratic political supervision, as required by the German Constitutional Court, thus creating conflict between monetary policies at the EU level and democracy at the state level, which in turn may further delegitimise the EU’s decisions in the eyes of the public (Schmitter and Lefkofridi 2016). Moreover, the current EU response to the growing COVID-19 crisis, shows that the Commission acts more as a supporter of the European Council and the intergovernmental equilibrium (Bressanelli and Chelotti 2016; Fabbrini and Puetter 2016), than as an independent actor capable of bolstering Brussels’ weak political centre.

The only way of overcoming this limitation and of implementing the political dimension of EU integration would entail a definitive weakening of the power and legitimacy of member states as a result of the complete supranationalization of core state powers, thus effectively achieving full system building for the EU (Bartolini 2005). The EU’s recent history clearly indicates that such an outcome is highly unlikely, with developments pointing to a partial retrenchment within national boundaries and to the victory of the intergovernmental approach over the supranational option (Fabbrini 2019). This model can only entail coordination among member states; it will not lead to implementation of the supranational option.

The national reaction to the broadening of the pervasiveness of core state powers has been characterised by the trans-nationalisation of the pro/anti-EU cleavage and the rise of Eurosceptic and nationalistic political entrepreneurs, capable of influencing national political agendas and of forcing mainstream parties in government to drop new plans for broader integration or to opt for incomplete, ineffective solutions (Jones et al. 2016) which result in a more highly-differentiated integration.

Supranational institutions’ lack of any autonomous extractive capacity, in particular in those sectors and policy fields that go beyond the traditional scope of the EU (Fabbrini and Puetter 2016), reveals the diminished power of the supranational system. This lack of power accounts for the diminished ability of the supranational institutions to play a leading role in the political response to crisis situations, leaving the substantial decisions to be taken by means of intergovernmental agreements. It is still early to assess whether the COVID-19 crisis may represent an effective opportunity to boost integration, or whether it will be remembered as a period of stalemate, of disintegration or of incomplete integration. What we can say is that we are not facing a Hamiltonian moment for the EU, or one of the effective empowerment of the supranational method. The different instruments established by the EU do not involve any mutualisation of EU member states’ inherited debts, and even the new common debt will not enjoy the benefit of joint-and-several guarantees. Furthermore, the main issue surrounding the EU plan, that is, how to repay the new debts, remains open to debate. National governments are unwilling to hand tax-raising powers to Brussels, and are sceptical about the Commission’s proposal to raise EU taxes to finance the recovery plan (Herszenhorn et al. 2020). Indeed, what we have at present is confirmation of the EU’s current inability to act as a political centre and its weak degree of legitimation in the eyes of its citizens, even in times of crisis.

## Footnotes

1 I refer to a structured coalition when there is a permanent coordination between a group of member states.
